# The Human CD4^+^ T Cell Response against Mumps Virus Targets a Broadly Recognized Nucleoprotein Epitope

**DOI:** 10.1128/JVI.01883-18

**Published:** 2019-03-05

**Authors:** Jelle de Wit, Maarten E. Emmelot, Martien C. M. Poelen, Josien Lanfermeijer, Wanda G. H. Han, Cécile A. C. M. van Els, Patricia Kaaijk

**Affiliations:** a Centre for Infectious Disease Control, National Institute for Public Health and the Environment, Bilthoven, The Netherlands; University of Kentucky College of Medicine

**Keywords:** cytotoxicity, mumps G5 outbreak strain, mumps infection, mumps patients

## Abstract

Mumps outbreaks among vaccinated young adults stress the need for a better understanding of mumps virus (MuV)-induced immunity. Antibody responses to MuV are well characterized, but studies on T cell responses are limited. We recently isolated a MuV-specific CD4^+^ T cell clone by stimulating peripheral blood mononuclear cells (PBMCs) from a mumps case with the viral nucleoprotein (MuV-N). In this study, we further explored the identity and relevance of the epitope recognized by the CD4^+^ T cell clone and *ex vivo* by T cells in a cohort of mumps cases. Using a two-dimensional matrix peptide pool of 15-mer peptides covering the complete MuV-N, we identified the epitope recognized by the T cell clone as MuV-N_110–124_ GTYRLIPNARANLTA, present in a well-conserved region of the viral protein. Upon peptide-specific stimulation, the T cell clone expressed the activation marker CD137 and produced gamma interferon, tumor necrosis factor, and interleukin-10 in a HLA-DR4-restricted manner. Moreover, the CD4^+^ T cells exerted a cytotoxic phenotype and specifically killed cells presenting MuV-N_110–124_. Furthermore, the identified peptide is widely applicable to the general population since it is predicted to bind various common HLA-DR molecules, and epitope-specific CD4^+^ T cells displaying cytotoxic/Th1-type properties were found in all tested mumps cases expressing different HLA-DR alleles. This first broadly recognized human MuV-specific CD4^+^ T cell epitope could provide a useful tool to detect and evaluate virus-specific T cell responses upon MuV infection or following vaccination.

**IMPORTANCE** Recent outbreaks of mumps among vaccinated young adults have been reported worldwide. Humoral responses against mumps virus (MuV) are well characterized, although no correlate of protection has been elucidated, stressing the need to better understand cellular MuV-specific immunity. In this study, we identified the first MuV T cell epitope, which is derived from the viral nucleoprotein (MuV-N) and was recognized by a cytotoxic/Th1 CD4^+^ T cell clone that was isolated from a mumps case. Moreover, the epitope was predicted to bind a broad variety of common HLA-DRB1 alleles, which was confirmed by the epitope-specific cytotoxic/Th1 CD4^+^ T cell responses observed in multiple mumps cases with various HLA-DRB1 genotypes. The identified epitope is completely conserved among various mumps strains. These findings qualify this promiscuous MuV T cell epitope as a useful tool for further in-depth exploration of MuV-specific T cell immunity after natural mumps virus infection or induced by vaccination.

## Abstract (precis)

Recent outbreaks of mumps among vaccinated young adults have been reported worldwide. Humoral responses against mumps virus (MuV) are well characterized, although no correlate of protection has been elucidated, stressing the need to better understand cellular MuV-specific immunity. In this study, we identified the first MuV T cell epitope, which is derived from the viral nucleoprotein (MuV-N) and was recognized by a cytotoxic/Th1 CD4^+^ T cell clone that was isolated from a mumps case. Moreover, the epitope was predicted to bind a broad variety of common HLA-DRB1 alleles, which was confirmed by the epitope-specific cytotoxic/Th1 CD4^+^ T cell responses observed in multiple mumps cases with various HLA-DRB1 genotypes. The identified epitope is completely conserved among various mumps strains. These findings qualify this promiscuous MuV T cell epitope as a useful tool for further in-depth exploration of MuV-specific T cell immunity after natural mumps virus infection or induced by vaccination.

## INTRODUCTION

The mumps virus (MuV) is an enveloped virus and belongs to the family of paramyxoviruses. Its nonsegmented, negative single-stranded RNA encodes seven proteins: nucleoprotein (N), phosphoprotein [(V/I/)P], matrix protein (M), fusion protein (F), small hydrophobic protein (SH), hemagglutinin-neuraminidase protein (HN), and large viral polymerase protein (L) (1). Humans are the only natural host for MuV. Clinically symptomatic MuV infection is characterized by parotid swelling, although complications such as orchitis, meningitis, and encephalitis can occur as well (2). Despite high vaccination coverage with live attenuated MuV vaccine, over the last decade several mumps outbreaks have been reported worldwide, in particular among vaccinated young adults (3, 4). This suggests the waning of vaccine-induced immunity.

MuV infection, as well as vaccination, induces a mumps-specific antibody response. Although an absolute immune correlate of protection against mumps does not exist, the level of neutralizing antibodies is considered to be indicative for protection (5). Antibodies directed against HN have been demonstrated to play a major role in neutralization of the virus (6–8). However, antibody concentrations against HN were relatively low or below detection levels after MuV infection or vaccination (9, 10). Interestingly, a large fraction of the mumps-specific antibodies is directed against N and can be detected in almost all individuals after vaccination (9–11). The mumps N is an abundant intraviral protein that plays an important role in transcription, replication, and virus budding (12). Several B cell epitopes within MuV-N have been described (10, 13), but despite being an immunodominant antigen, the concentrations of N-specific antibodies do not correlate with the virus-neutralizing capacity of serum from vaccinees (10) or from individuals with a history of mumps infection (9). In contrast to the antibody response, the cellular arm of the immune response against MuV, and in particular T cell responses, is less well characterized. CD4^+^ T cells are important in viral infections, since they provide help to B cells in enhanced antibody responses, and CD4^+^ T cells help CD8^+^ T cells in cytotoxic responses and memory differentiation (14).

Since MuV-N was recently confirmed to be an immunodominant antigen for antibody responses after vaccination of young adults (10), it can be anticipated to play an essential role in MuV-induced CD4^+^ T cell immunity as well. N-encoded T cell epitopes were described for several other viruses, including measles (15, 16), rabies (17), and influenza viruses (18). Thus far, not a single MuV T cell epitope has been identified that has been confirmed with a functional T cell assay.

We recently isolated a MuV-specific CD4^+^ T cell clone, named MuTER.1, by stimulating PBMCs of a mumps case with the viral nucleoprotein (MuV-N). Here, we identified the first naturally processed human CD4^+^ T cell epitope of MuV. The CD4^+^ T cell clone presented a cytotoxic/Th1-type profile and was able to specifically kill cells presenting this epitope in the context of HLA-DRB1*04. Furthermore, cytotoxic/Th1-type CD4^+^ T cell responses against this epitope were found in a panel of mumps cases expressing other HLA-DR alleles. This promiscuous HLA-DR-restricted MuV T cell epitope seems to represent an striking immunodominance in the MuV cellular immune response and could be a useful tool to detect and characterize in depth MuV-specific human CD4^+^ T cell responses following mumps infection and vaccination.

## RESULTS

### Generation and selection of a MuV-N-specific T cell clone.

**FIG 1:**
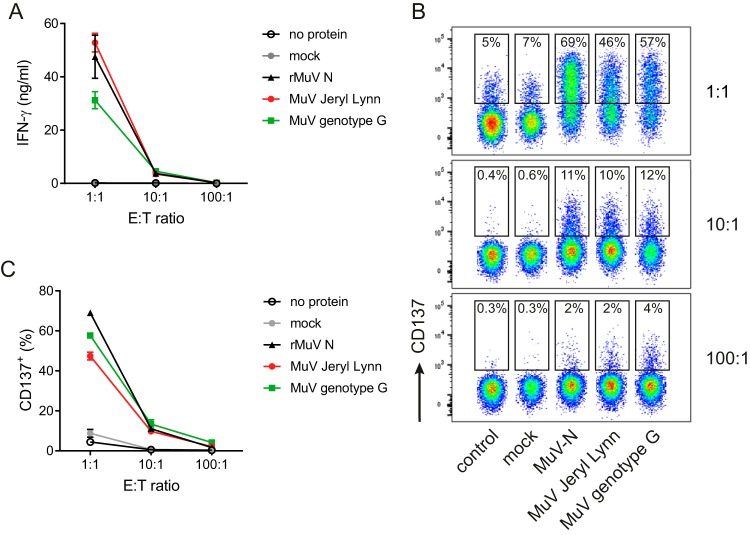
The CD4^+^ T cell clone is specific for MuV. MuTER.1 T cells were cocultured at a 1:1, 10:1, or 100:1 effector/target (E:T) ratio, as indicated, with autologous BLCL that were pulsed with no protein, mock, recombinant N, live MuV JL, or genotype G. After 20 h, T cell responses were analyzed by measuring the levels of secreted IFN-γ in the supernatant (A) or by staining for the activation marker CD137 (B and C). Data shown are from three individual experiments, with means ± the standard deviations (SD), and flow cytometry data are from one representative experiment (C). The T cell response, measured by IFN-γ secretion or CD137 expression (A and B), was significantly higher after stimulation with recombinant N, live MuV JL, or genotype G compared to stimulation with no protein or mock at all effector/target ratios (*P* < 0.0001).

MuV-N-specific T cell clones were generated from PBMCs taken from a 24-year-old female 35 days following onset of symptomatic mumps disease, by stimulation with rMuV-N and subsequent limiting dilution. Eight MuV-specific T cell clones were successfully generated, and all were of CD4^+^ T cell lineage (data not shown). The most potent CD4^+^ T cell clone was selected based on expansion and activation by antigen stimulation. This CD4^+^ T cell clone, named MuTER.1, not only responded to autologous BLCL that were pulsed with the rMuV-N, but also produced gamma interferon (IFN-γ) and expressed the T cell activation marker CD137 upon stimulation with Epstein-Barr virus-transformed B-lymphoblastoid cell lines (BLCL) that were infected with either live vaccine virus (JL) or the outbreak strain (genotype G) (Fig. 1). These data demonstrate that MuTER.1 recognizes a naturally processed MuV-N peptide that is present in both the vaccine and outbreak strain. In addition, the clonal nature of MuTER.1 was confirmed by the CDR3β usage, since 98.6% of the sequences showed the same CDR3β usage of TRBV19 and TRBJ2-7. Interestingly, the mRNA expression of two different alpha-chains showed that 80% of the cells expressed TRAV13-2 and TRAJ39 gene segments (data not shown).

### Identification of MuV epitope recognized by MuTER.1.

**FIG 2:**
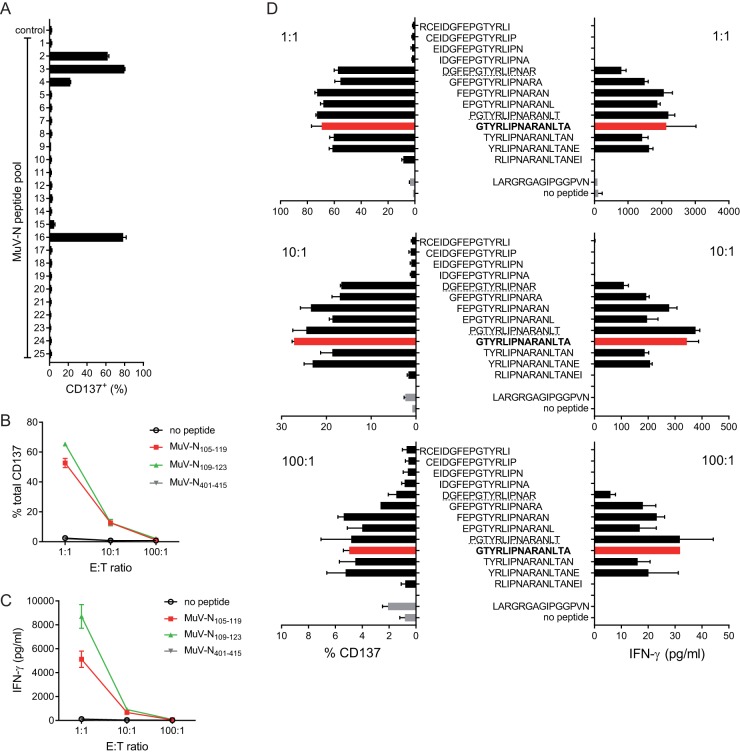
MuTER.1 clone responds to peptides with the core sequence YRLIPNAR. MuTER.1 cells were stimulated by peptide-pulsed autologous BLCL. (A) After 6 h, T cell activation by 25 different peptide pools was determined by expression of CD137 of CD4^+^ T cells, in a single experiment. (B and C) BLCL were pulsed with peptides MuV-N_105–119_ or MuV-N_109–123_ (from pools 2, 3, and 16) or a nonstimulating control peptide MuV-N_401–415_. Clone MuTER.1 was stimulated at a 1:1, 10:1, or 100:1 ratio, as indicated, with pulsed BLCL, and T cell activation was determined from the expression of CD137 (B) or IFN-γ secretion (C). (D) MuTER.1 cells were stimulated with BLCL pulsed with 15-mer peptides representing the MuV-N_101–127_ sequence with 14-mer amino acid overlap in 1:1, 10:1 or 100:1 ratio, as indicated. Peptides MuV-N_105–119_ or MuV-N_109–123_ are underlined. The red bars present the 15-mer peptide MuV-N_110–124_, GTYRLIPNARANLTA, that was selected for further characterization of the MuTER.1 clone. Data shown are triplicates, with means ± the SD, from one representative experiment of two to three individual experiments. The MuTER.1 clone showed significantly higher CD137^+^ expression upon stimulation with peptide pools 2, 3, 4, and 16 compared to medium control (*P* < 0.0001). The T cell response, measured by CD137 expression or IFN-γ secretion (B and C), was significantly higher after stimulation with peptides MuV-N_105–119_ (*P* < 0.0001) or MuV-N_109–123_ (*P* < 0.001) compared to stimulation with no peptide or control peptide MuV-N_401–415_, at an E:T of 1:1 and 1:10.

Using an overlapping set of synthetic peptides spanning the whole MuV-N, the epitope recognized by the MuTER.1 clone was assessed. For this purpose, autologous BLCL were pulsed with the various peptide pools, and their capacity to activate MuTER.1 was determined by measuring CD137 expression with flow cytometry (Fig. 2A). Of the 25 peptide pools, 3 (pools 2, 3, and 16) induced strong activation of MuTER.1, and 1 pool (catalog no. 4) induced moderate T cell activation, indicating that the epitope recognized by the T cell clone was present within these peptide pools (Fig. 2A). Two individual peptides, MuV-N_105-119_ and MuV-N_109-123_, were deduced from the positive peptide pools. Subsequently, stimulation with these two individual 15-mer peptides resulted in a positive response of the T cell clone, confirming the presence of the epitope within these peptides, but not a control peptide MuV-N_401–415_ (Fig. 2B and C). To determine the optimal 15-mer that accounted for a positive response of MuTER.1, a new set of 15-mer peptides with a 14-mer amino acid overlap around the region of the positive peptides (MuV-N_101-127_) was subsequently tested. Stimulation with peptide-pulsed BLCL revealed that MuTER.1 responded to peptide in the range MuV-N_105–126_ (Fig. 2D), with YRLIPNAR as the core sequence. For further characterization of the MuTER.1 clone, we used the 15-mer peptide MuV-N_110–124_, GTYRLIPNARANLTA (here named GTYR).

### HLA restriction to HLA-DRB1*04.

**FIG 3:**
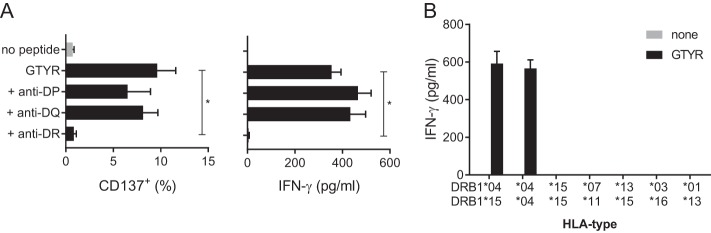
GTYR-specific activation of MuTER.1 is restricted by HLA-DR*04. (A) MuTER.1 cells were stimulated with autologous BLCL pulsed with GTYR peptide in the presence of anti-HLA-DR, anti-HLA-DQ, or anti-HLA-DP blocking antibodies. T cell activation was determined by expression of CD137 and IFN-γ secretion in the supernatant, as indicated. (B) BLCL with various HLA-DRB1 types as indicated were pulsed with GTYR peptide. T cell activation by these peptide-loaded BLCL was measured by IFN-γ secretion in the supernatant. Left bar represents autologous BLCL. T cell response, measured by CD137 expression or IFN-γ secretion (A), was significantly blocked with anti-HLA-DR antibodies (*P* < 0.01) and not with anti-HLA-DQ or anti-HLA-DP antibodies.

In order to determine the HLA class II restricting element of MuTER.1, clone cells were stimulated with GTYR peptide pulsed autologous BLCL in the presence of HLA-DR-, HLA-DQ-, or HLA-DP-blocking antibodies. Although blocking of HLA-DQ or HLA-DP molecules did not influence T cell activation, blocking HLA-DR molecules completely abolished the IFN-γ and CD137^+^ T cell response (Fig. 3A).

More specifically, the MuTER.1 clone recognized the GTYR peptide in a HLA-DRB1*04-restricted manner, which became evident after measuring IFN-γ release upon GTYR peptide stimulation with BLCL as antigen-presenting cells sharing or lacking specific HLA-DR alleles with the donor of MuTER.1 clone (Fig. 3B).

### MuTER.1 has cytotoxic properties and specifically kills cells presenting GTYR peptide.

**FIG 4:**
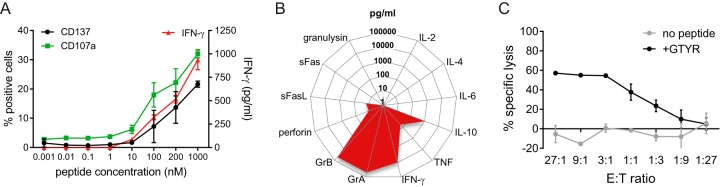
MuV-specific CD4^+^ T cell clone MuTER.1 display a cytotoxic/Th1 phenotype. MuTER.1 cells were stimulated by autologous BLCL that were pulsed with GTYR peptide for activation and functional analysis. (A) T cell activation to a dose range of GTYR peptide was determined by IFN-γ secretion in the supernatant, and expression of the activation marker CD137 or degranulation marker CD107a. (B) Levels of secreted inflammatory and cytotoxicity-associated molecules were determined in the supernatant. Background cytokine levels in MuTER.1 cocultures with nonpulsed BLCL were subtracted. The levels of the cytokines indicated are averages of triplicate wells. (C) BLCL were pulsed with GTYR peptide and cocultured with MuTER.1 at various E:T ratios, as indicated. Killing of BLCL was determined after 24 h by PI staining.

Next, the MuTER.1 clone was stimulated with decreasing concentrations of the GTYR peptide to determine the threshold level for activation. After peptide stimulation, MuTER.1 produced IFN-γ and expressed CD137 in a similar dose-response pattern, i.e., with minimal T cell activation at a peptide concentration of ≤10 nM, which increased at higher doses (Fig. 4A). Interestingly, MuTER.1 expressed the degranulation marker CD107a upon stimulation with the GTYR peptide (Fig. 4A). This implies that the CD4^+^ T cells from MuTER.1 can exert cytotoxic functions, which was further demonstrated by analysis of multiple parameters associated with cytotoxicity (Fig. 4B). Peptide stimulation of MuTER.1 resulted in increased levels of granzyme A and B, perforin and soluble Fas ligand (sFasL), as well as IFN-γ, tumor necrosis factor (TNF), and interleukin-10 (IL-10). The cytotoxic capacity was confirmed in a cell killing assay, where MuTER.1 clone showed to be able to lyse autologous BLCL that were pulsed with the GTYR peptide (Fig. 4C). Of the seven other MuV-specific T cell clones that were generated, four responded to GTYR peptide stimulation as well and displayed a similar, multifunctional phenotype by secretion of IFN-γ, IL-10, granzyme A, and granzyme B (data not shown).

### GTYR-directed cytotoxic/Th1 type T cell responses are detected in mumps cases in the context of a broad range of HLA-DR alleles.

**FIG 5:**
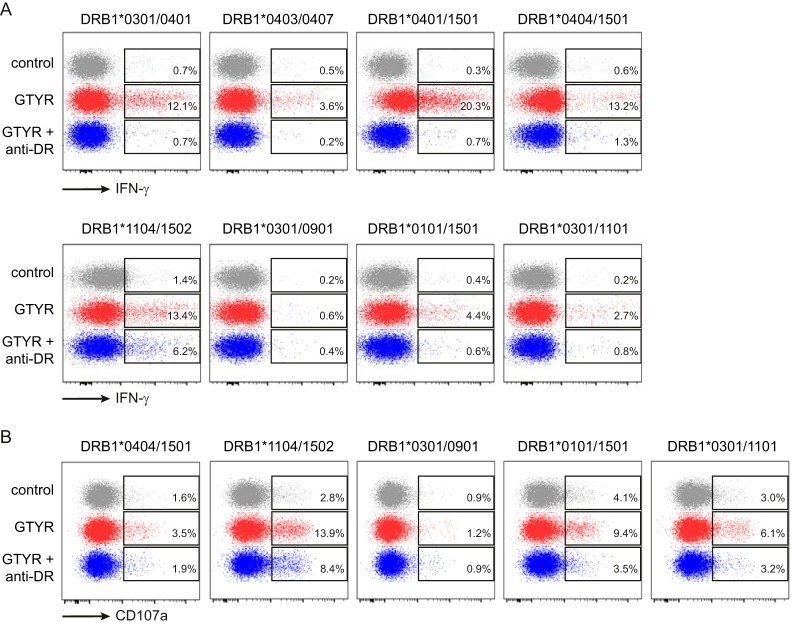
GTYR-specific CD4^+^ T cell responses in multiple mumps cases with different HLA-DR types. PBMCs from different mumps cases (1 to 2 months after clinical onset) with known HLA-DRB1 types, as indicated, were stimulated with GTYR peptide for 12 days in the presence of IL-2 to expand GTYR-specific CD4^+^ T cells. After 12 days, cells were rested for 24 h and restimulated with GTYR peptide in absence or presence of anti-HLA-DR blocking antibodies. GTYR-specific CD4^+^ T cells were determined after 6 h by intracellular cytokine staining for IFN-γ (A) and CD107a (B) surface expression. The data are from two experiments with eight and five different mumps cases, respectively.

Next, the clinical relevance was investigated by evaluating GTYR peptide T cell responses in mumps cases with HLA-DRB1*04 type and in mumps cases with alternative HLA-DRB1 types. To be able to detect measurable numbers of specific T cells, peripheral blood mononuclear cells (PBMCs) of eight mumps cases, of which one was homozygous and three were heterozygous for HLA-DRB1*04, were stimulated with the GTYR peptide in the presence of IL-2, and CD4^+^ T cell responses, as well as their cytotoxic phenotypes, were characterized after a 12-day expansion. All four mumps patients expressing an HLA-DRB1*04 molecule showed a good CD4^+^ T cell response against the GTYR peptide, including the mumps case from which the clone was derived from (HLADRB1*04/1501) (Fig. 5A, top panel). Moreover, three of four mumps cases that were negative for HLA-DRB1*04 also displayed a significant mumps-specific CD4^+^ T cell response upon stimulation with GTYR peptide (Fig. 5A, bottom panel). A fourth HLA-DRB1*04-negative mumps case (DR*0301/DR*0901) also seemed to show a mumps-specific T cell response, but the frequency of IFN-γ-expressing cells was low. In line with data of the MuTER.1 clone, day 13 stimulated T cells from five GTYR responders that were tested all displayed cytotoxic properties by expression of the degranulation marker CD107a (Fig. 5B). Blocking of the HLA-DR molecules suppressed the peptide-specific T cell response in all cases, confirming HLA-DR-restricted responses (Fig. 5A and B, blue plots). One mumps case (with the DR*1104/DR*1502 haplotype) still showed enhanced IFN-γ and CD107a expression upon HLA-DR blocking, although the expression was reduced by 40 to 50%, indicating that the peptide-specific T cell response was only partly HLA-DR restricted in this patient.

### GTYR can be presented by a broad range of HLA-DR alleles.

In view of the wide recognition of the GTYR epitope, we evaluated whether the MuV-N_110–124_ sequence contained a hot spot of HLA-DR binding motifs. *In silico* binding predictions were performed on the whole MuV-N sequence, with a special focus on the GTYR peptide region, for 17 different HLA-DRB1 alleles, including the 10 most common HLA alleles worldwide (19), as well as those of the selected mumps cases. Our identified epitope GTYRLIPNARANLTA was predicted to be one of the strongest and most promiscuous HLA-DR binders, i.e., for 8 of 17 HLA-DR types the binding potential of GTYRLIPNARANLTA was strong (see Fig. S1 in the supplemental material). Other potential binding regions, predicted by the *in silico* binding capacity of linear overlapping 15-mer peptides representing the entire MuV-N protein sequence from different MuV strains, are visualized in Fig. S1. When searching for a promiscuous pan-DR binding motif within the MuV-N_110-124_ 15-mer sequence, an anchor-site-rich core 9-mer sequence could be identified, MuV-N_112–120_ YRLIPNARA. Y (tyrosine) at the position 1 (P1), I (isoleucine) at P4, N (asparagine) at P6, and A (alanine) at P7 and P9 have been identified as anchors or auxiliary anchors for various HLA-DR alleles, including all HLA-DR alleles from the eight mumps cases.

Moreover, apart from being promiscuous, the epitope appeared to be conserved across various relevant mumps strains, including the vaccine strain, as well as genotype G and H strains (Fig. S1 and S2), which caused outbreaks in vaccinated populations (3, 20). Collectively, these data indicate redundancy in HLA-DR presentation and broad recognition of the GTYR epitope in the population.

## DISCUSSION

The global reemergence of mumps in vaccinated young adults emphasizes the need for more insight in the vaccine-induced immunity. Waning of antibody levels has been associated with mumps vaccine failure (3, 4, 21), but we recently found that suboptimal cellular immune responses can contribute to this as well (22). We and others have reported that T cell responses are lower or less functional in vaccinees than in naturally infected persons (22–24). However, only limited studies on mumps-specific T cell immunity are available.

The role of CD4^+^ T cells in viral infections is evident, as they provide help to B cells for an enhanced antibody response, and CD4^+^ T cells help the generating cytotoxic and memory CD8^+^ T cells (14). The mumps vaccine-induced cellular response seems more persistent compared to the induced humoral response (25). Several studies show IFN-γ production by MuV-stimulated PBMCs, or by (CD4^+^ CD69^+^) T cells after vaccination, but the frequencies are low (23–25). In an earlier study, we showed that strong polyfunctional CD8^+^ T cell responses persisted up to 3 years in naturally infected persons, while suboptimal CD8^+^ T cell responses were found in recently vaccinated children. A key factor here might be suboptimal CD4^+^ T cell help. At this moment, the role of CD4^+^ T cells in immunity against MuV is poorly understood, and identification of T cell epitopes may help to characterize the MuV-specific T cell response. Here, we identified the first MuV-specific CD4^+^ T cell epitope, i.e., MuV-N_110–124_ sequence GTYRLIPNARANLTA, which was targeted by the MuV-specific CD4^+^ T cell clone, MuTER.1, isolated from a mumps case. Upon GTYR peptide-specific stimulation, the MuTER.1 clone produced IFN-γ and showed a cytotoxic effector phenotype by expression of CD107a. The cytotoxic phenotype was further confirmed by the capacity of MuTER.1 cells to release cytotoxic proteins as granzyme A/B and by the ability to specifically kill cells presenting the GTYR peptide.

Interestingly, in a panel of mumps cases tested 1 to 2 months after onset of clinical symptomatic mumps, all donors tested had GTYR-specific IFN-γ-producing and/or CD107a-expressing CD4^+^ T cells. Whether the identified epitope thus preferentially induces cytotoxic/Th1 type CD4^+^ T cells, or whether the effector phenotype induced is rather a matter of how the specific T cells were primed, for example after MuV infection or after vaccination, remains to be elucidated.

Flynn and Mahon reported that murine CD4^+^ T cell lines generated by attenuated MuV immunization produced either IFN-γ or IL-10 (26), but their cytotoxic capacity was not determined. IFN-γ and IL-10 production was also seen upon stimulation of total PBMCs from naturally infected and vaccinated persons with MuV lysate (23). Next to its cytotoxic phenotype and capacity to secrete IFN-γ, the MuTER.1 CD4^+^ T cell clone predominantly produced TNF and IL-10, suggesting a multifunctional, inflammatory phenotype. IFN-γ can promote (cellular) clearance of the virus (27), whereas IL-10 may help to maintain the humoral response (28). Cytotoxicity through FasL, perforin, and granzyme release may help to clear the infection and downregulate the response by killing of epitope-presenting cells (29, 30).

Although the MuTER.1 clone recognized the GTYR peptide via HLA-DRB1*04, this was not the only HLA-DR-restricting element. Significant T cell responses could also be induced in PBMCs from mumps cases with other HLA-DRB1 genotypes. This was further supported by *in silico* prediction, showing a strong binding affinity of this peptide to many other HLA-DRB1 types. Moreover, within the MuV-N sequence, our identified epitope was predicted to be one of the most potent epitopes with high binding affinity for multiple HLA-DRB1 types. In addition, the GTYR epitope is conserved among the (recently) circulating wild-type MuV strains, implying cross-reactivity to multiple MuV strains. Also, it would be interesting to map more naturally processed MuV-specific epitopes with broad population coverage in order to better understand the T cell responses following natural infection or vaccination.

Taken together, we identified the first naturally processed human MuV epitope that is broadly and strongly recognized in a HLA-DR-restricted manner by cytotoxic/Th1 type CD4^+^ T cells from persons recently infected with mumps. Since this epitope is highly conserved among various mumps strains and has a broad coverage of HLA-DRB1 types, it may be a potent epitope to explore in depth the properties of MuV-specific T cells that can provide clues for vaccine failure and/or vaccine development.

## MATERIALS AND METHODS

### Subjects and peripheral blood sampling.

Peripheral blood samples of individuals that were recently infected by MuV, here named mumps cases, were collected 1 to 2 months after disease onset as part of the observational clinical study VAC-263 (NL37852.094). All mumps cases were clinically and laboratory confirmed (31). Peripheral blood from healthy donors was obtained from the Sanquin Blood Supply. Written informed consent was obtained for all sample collections and subsequent laboratory analyses. The study was approved by the medical ethical committee and performed in accordance with the Declaration of Helsinki.

### PBMC isolation and BLCL generation.

PBMCs from mumps cases and healthy donors were isolated by centrifugation on a Ficoll-Hypaque gradient (Pharmacia Biotech) and cryopreserved (in 90% fetal calf serum–10% dimethyl sulfoxide) at −135°C until use. Epstein-Barr virus-transformed B-lymphoblastoid cell lines (BLCL) were generated as described previously (32).

### Virus strains and peptides.

Generation of the MuV stock Jeryl Lynn vaccine strain (Mumpsvax; Merck) and the genotype G outbreak strain were as described before (5, 32). Briefly, the supernatant of MuV-inoculated Vero cells was harvested at peak cytopathic effect, centrifuged (485 × *g*), and filtered (5 μm pore size). Virus stocks were aliquoted and stored at –80°C until use. Supernatant of uninfected Vero cells was used as a mock control after freeze-thawing cycles to lyse the cells analogous to MuV-infected cells.

15-mer peptides with an 11-amino-acid overlap spanning the entire MuV-N protein sequence were synthesized (JPT) for MuV-N of the Jeryl Lynn (JL) vaccine-strain (GenBank accession no. AAL83738.1) and for the genotype G strain (GenBank accession no. AFO62178.1 [G5 New York.USA/40.09/1]). A two-dimensional matrix of 25 different pools of 12 to 13 peptides each was prepared to identify the peptide specificity of the MuV-N-specific T cell clone. For MuV-N_102–126_, 15-mer peptides with a 14-amino-acid overlap were synthesized (PepScan).

### T cell clone generation.

PBMCs from an HLA-typed MuV case were thawed and subsequently cultured in complete RPMI medium (i.e., RPMI 1640 [Life Technologies] containing 10% human AB serum [Sigma], and 100 U/ml penicillin, 100 μg/ml streptomycin, and 292 μg/ml l-glutamine [Gibco]). At days 0 and 7, cells were stimulated with 1 μg/ml recombinant MuV nucleoprotein (rMuV-N; Prospec), and 1 ng/ml recombinant human IL-2 (Miltenyi Biotec) was added on days 5, 8, and 11. Next, the cells were plated at 0.3 cells per well in round-bottom 96-well plates and stimulated with irradiated autologous BLCL that were pulsed with rMuV-N at 1 μg/ml to generate T cell clones, including MuTER.1. IL-2 (5 ng/ml) was added every 3 to 4 days. T cell clones were subsequently propagated for at least two rounds with a feeder mixture consisting of irradiated feeder PBMCs from three random healthy adult blood donors, irradiated BLCL from two unrelated donors, 1 μg/ml phytohemagglutinin (Sigma), and 5 ng/ml IL-2. Extra IL-2 (5 ng/ml) was added every 3 to 4 days. To confirm the clonal nature of the MuTER.1 clone, clonotype analysis was performed as described previously with minor modifications (33). Briefly, mRNA from 500,000 cells of the clone was extracted with a NucleoSpin RNA kit (Macherey-Nagel) according to the manufacturer’s instructions. Anchored template-switch reverse transcription-PCR (RT-PCR) was performed to amplify all expressed TCRα and TCRβ chains linearly. PCR products were isolated from a 1% agarose gel with a Gel and PCR Clean-Up kit (Macherey-Nagel) according to the manufacturer’s instructions. Sequencing and library preparation were conducted on an Illumina MiSeq sequencer. Sequences were analyzed using the RTCR software tool (34). Expanded T cell clones were cryopreserved at −135°C until use.

### T cell activation analysis.

In experiments with MuTER.1 cells, autologous BLCL were cultured for 24 h in complete RPMI with live MuV at an MOI of 2 (JL vaccine strain or genotype G outbreak strain), 1 μg/ml rMuV-N, or mock, or BLCL were pulsed with 1 μM peptide for 2 h. After the BLCL cells were washed thoroughly, they were added in different ratios as indicated to the CD4^+^ T cell clone MuTER.1 (20,000 T cells per well in a 96-well round-bottom plate, triplicates per stimulation condition) and cultured in AIM-V (Gibco) supplemented with 2% human AB serum at 37°C and 5% CO_2_ for 24 h. Where indicated, BLCL were incubated with 4 μg/ml anti-HLA-DR (clone B8.112; in-house), anti-HLA-DQ (clone SPVL3; in-house), or anti-HLA-DP (clone B7/21; Leinco Technologies) blocking antibodies prior to coincubation with T cells. Readout for T cell activation was determined by flow cytometry assays or cytokine secretion analysis.

To determine GTYR-specific CD4^+^ T cells in mumps cases, 2 × 10^6^ PBMCs were cultured in AIM-V with 2% human AB serum and stimulated at days 0 and 6 with 1 μM GTYR peptide. IL-2 (2.5 ng/ml) was added every 3 days. Cells were harvested at day 12, rested for 24 h in AIM-V with 2% human AB serum, and then restimulated for 6 h with or without 1 μM GTYR peptide; brefeldin A and monensin (BD) were added during the last 4 h of culture. Restimulated 13-day cultures were subsequently stained for flow cytometry analysis.

### Flow cytometry.

Cells were labeled with fluorochrome-conjugated antibodies, anti-CD3 APC-R700, anti-CD4 BV711, anti-CD8 BV786, anti-CD56 BV510, and anti-CD19 APC and with fixable viability stain eF780 (all BD). Anti-CD107a BV421 (BioLegend) was added during culture. For intracellular staining, cells were fixed, permeabilized, and stained with phycoerythrin-conjugated anti-CD137 and allophycocyanin-conjugated anti-IFN-γ (BD) using the Foxp3/transcription factor staining buffer set (eBioscience) according to the manufacturer’s protocol. Cells were acquired on an LSRFortessa X20 flow cytometer (BD). Data were analyzed using FlowJo (v10; Tree Star). The results are expressed as the percentage of CD137^+^ or CD107a^+^ cells in the CD3^+^ CD4^+^ live gate.

### HLA typing and HLA restriction of MuTER.1.

Blood cell samples from mumps cases were molecularly typed at four digits for the HLA-DRB1, HLA-DQB1, and HLA-DPB1 loci at high resolution by next-generation sequencing (LTI, University Medical Center, Utrecht, The Netherlands). Cells from the mumps case who was donor of the MuTER.1 CD4^+^ T cell clone was HLA genotyped as HLA-DRB1*04:01, HLA-DRB1*15:01, HLA-DQB1*03:02, HLA-DQB1*06:02, HLA-DPB1*20:01, and HLA-DPB1*75:01. Since this MuTER.1 donor was typed as HLA-DRB1*04:01 and HLA-DRB1*15:01 positive and because of linkage disequilibrium of the DRB1*04 and DRB1*15 haplotypes with functional HLA-DRB4*01 and HLA-DRB5*01 genes, respectively, HLA-DR51 and HLA-DR53 molecules are coexpressed. To map HLA-DR restriction of MuTER.1 T cells to one of the four expressed HLA-DR molecules, allogeneic HLA-typed BLCL were used with the following HLA-DR types: homozygous for DRB1*04/DRB4*01 or DRB1*15/DRB5*01; heterozygous for DRB1*07/DRB4*01/DRB1*11, DRB1*13/DRB1*15/DRB5*01, or DRB1*03/DRB1*16/DRB5*01; and DRB1*01/DRB1*13 (negative control).

### Cytokine secretion analysis.

To determine cytokine release in stimulated T cell cultures, cell-free culture supernatant was used in standard sandwich enzyme-linked immunosorbent assay kits for IFN-γ (eBioscience) or in a multiplex bead-based assay quantitating levels of IL-2, IL-4, IL-6, IL-10, TNF, IFN-γ, granzyme A (GrA), granzyme B (GrB), perforin, soluble Fas ligand (sFasL), soluble Fas (sFas), and granulysin (LEGENDplex human CD8/NK panel; BioLegend) according to the manufacturers’ instructions and using FACSCanto (BD).

### Flow cytometry-based killing assay.

Autologous BLCL were labeled with CellTrace violet (Life Technologies) and pulsed for 2 h with 1 μM peptide (GTYRLIPNARANLTA) or without peptides as control. MuTER.1 (20,000 cells per well) and BLCL were coincubated at different effector/target ratios in AIM-V supplemented with 2% human AB serum. Flow-count fluorospheres (Beckman Coulter) and propidium iodide (PI) were added after 24 h, and counts of viable (PI-negative) BLCL cells were acquired immediately using FACSCanto. Specific lysis was calculated as follows: % specific lysis = [(counts of control viable target cells) – (counts of peptide-stimulated viable target cells)]/[counts of control viable target cells] × 100%.

### HLA class II binding prediction of MuV-N.

The pan-specific prediction method NETMHCIIpan3.2 was used to *in silico* determine binding sites within MuV-N for different HLA-DRB1 alleles (35). The predicted binding to the most abundant HLA-DRB1 types was determined for 15-mers spanning the total MuV-N sequence of several mumps strains: vaccine strains JL2 (GenBank AAL83738.1) and JL5 (GenBank AAK83227.1), outbreak strains genotype G genotype NewYork/40.09 (GenBank AFO62178.1) and Iowa/06 (GenBank AFO62133.1), and outbreak strain genotype H Virginia/10.12 (GenBank ARL00360.1). A threshold for strong binding peptides was set on % rank < 2; The % rank defines how the predicted affinity for a given peptide ranks compared to a set of 200,000 random natural peptides of the same length. The NetMHCIIpan database (35) was used to identify shared anchor residues or auxiliary anchors for different HLA-DRB1 alleles within amino acid positions P1 through P9 of the MuV-N_112–120_ YRLIPNARA core epitope.

### Statistical analyses.

Data were analyzed with GraphPad Prism using *t* tests, and a *P* value of <0.05 was considered significant.

## Supplementary Material

Supplemental file 1
