# Supplementary material for: The Human CD4+ T Cell Response against Mumps Virus Targets a Broadly Recognized Nucleoprotein Epitope
Source: J Virol. 2019 Mar 5;93(6):e01883-18. doi: 10.1128/JVI.01883-18 (PMC6401470; doi:10.1128/JVI.01883-18)
Supplement: Supplemental file 1 [file JVI.01883-18-s0001.pdf]

SUPPLEMENTARY FIGURE LEGENDS

Figure S1. *In silico* prediction of peptide binding to most abundant HLA-DR alleles.

The pan-specific prediction method NETMHCIIpan3.2 was used to *in silico* determine binding sites within MuV-N for HLA-DR alleles that are most abundant among the Caucasian population. Shown is per HLA-DR allele the % Rank per tested 15-mer. Positions of peptide sequences considered strong binders, with a % Rank < 2, are recognized as intersecting the green cut-off zone.

Figure S2. Alignment of MuV-N amino acid sequences of various mumps strains.

Blue boxes indicate differences between MuV strains. Vaccine strains: JL2, Jeryl Lynn 2; JL5, Jeryl Lynn 5 (vaccine strains). Recent outbreak strains: genG_NY, genotype G NewYork strain; genG_Iowa, genotype G Iowa strain; genH, genotype H strain. The conserved MuV-NP$_{110\text{-}124}$ MuTER.1 epitope sequence GTYRLIPNARANLTA is indicated in red.

Figure S1

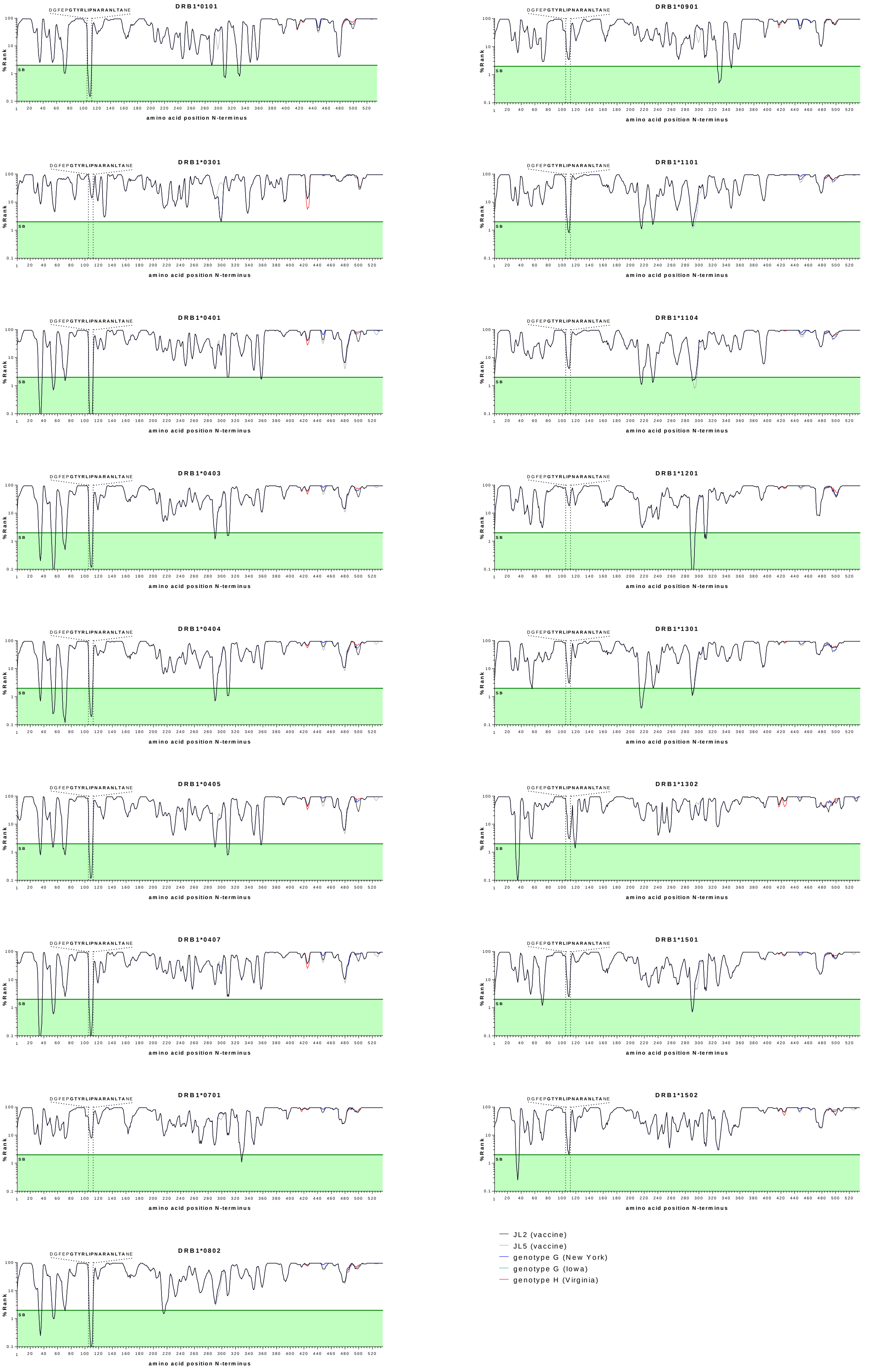
DRB1*0101
DRB1*0901
DRB1*0301
DRB1*1101
DRB1*0401
DRB1*1104
DRB1*0403
DRB1*1201
DRB1*0404
DRB1*1301
DRB1*0405
DRB1*1302
DRB1*0407
DRB1*1501
DRB1*0701
DRB1*1502
DRB1*0802
DGFEPGTYRLIPNARANLTANE
% Rank
SB
amino acid position N-terminus
JL2 (vaccine)
JL5 (vaccine)
genotype G (New York)
genotype G (Iowa)
genotype H (Virginia)

Figure S2

JL2	M L S V L K A F E R F T I E Q E L Q D R G E E G S I P P E T L K S A V K V F V I N T P N P T T R Y Q M L N F C L R I I C	60
JL5	M S S V L K A F E R F T I E Q E L Q D R G E E G S I P P E T L K S A V K V F V I N T P N P T T R Y Q M L N F C L R I I C	60
genG_NY	M S S V L K A F E R F T I E Q E L Q D R G E E G S I P P E T L K S A V K V F V I N T P N P T T R Y Q M L N F C L R I I C	60
genG_Iowa	M S S V L K A F E R F T I E Q E L Q D R G E E G S I P P E T L K S A V K V F V I N T P N P T T R Y Q M L N F C L R I I C	60
genH	M S S V L K A F E R F T I E Q E L Q D R G E E G S I P P E T L K S A V K V F V I N T P N P T T R Y Q M L N F C L R I I C	60
JL2	S Q N A R A S H R V G A L I T L F S L P S A G M Q N H I R L A D R S P E A Q I E R C E I D G F E P **G T Y R L I P N A R A**	120
JL5	S Q N A R A S H R V G A L I T L F S L P S A G M Q N H I R L A D R S P E A Q I E R C E I D G F E P **G T Y R L I P N A R A**	120
genG_NY	S Q N A R A S H R V G A L I T L F S L P S A G M Q N H I R L A D R S P E A Q I E R C E I D G F E P **G T Y R L I P N A R A**	120
genG_Iowa	S Q N A R A S H R V G A L I T L F S L P S A G M Q N H I R L A D R S P E A Q I E R C E I D G F E P **G T Y R L I P N A R A**	120
genH	S Q N A R A S H R V G A L I T L F S L P S A G M Q N H I R L A D R S P E A Q I E R C E I D G F E P **G T Y R L I P N A R A**	120
JL2	**N L T A** N E I A A Y A L L A D D L P P T I N N G T P Y V H A D V E G Q P C D E I E Q F L D R C Y S V L I Q A W V M V C K	180
JL5	**N L T A** N E I A A Y A L L A D D L P P T I N N G T P Y V H A D V E G Q P C D E I E Q F L D R C Y S V L I Q A W V M V C K	180
genG_NY	**N L T A** N E I A A Y A L L A D D L P P T I N N G T P Y V H A D V E G Q P C D E I E Q F L D R C Y S V L I Q A W V M V C K	180
genG_Iowa	**N L T A** N E I A A Y A L L A D D L P P T I N N G T P Y V H A D V E G Q P C D E I E Q F L D R C Y S V L I Q A W V M V C K	180
genH	**N L T A** N E I A A Y A L L A D D L P P T I N N G T P Y V H A D V E G Q P C D E I E Q F L D R C Y S V L I Q A W V M V C K	180
JL2	C M T A Y D Q P A G S A D R R F A K Y Q Q Q G R L E A R Y M L Q P E A Q R L I Q T A I R K S L V V R Q Y L T F E L Q L A	240
JL5	C M T A Y D Q P A G S A D R R F A K Y Q Q Q G R L E A R Y M L Q P E A Q R L I Q T A I R K S L V V R Q Y L T F E L Q L A	240
genG_NY	C M T A Y D Q P A G S A D R R F A K Y Q Q Q G R L E A R Y M L Q P E A Q R L I Q T A I R K S L V V R Q Y L T F E L Q L A	240
genG_Iowa	C M T A Y D Q P A G S A D R R F A K Y Q Q Q G R L E A R Y M L Q P E A Q R L I Q T A I R K S L V V R Q Y L T F E L Q L A	240
genH	C M T A Y D Q P A G S A D R R F A K Y Q Q Q G R L E A R Y M L Q P E A Q R L I Q T A I R K S L V V R Q Y L T F E L Q L A	240
JL2	R R Q G L L S N R Y Y A M V G D I G K Y I E N S G L T A F F L T L K Y A L G T K W S P L S L A A F T G E L T K L R S L M	300
JL5	R R Q G L L S N R Y Y A M V G D I G K Y I E N S G L T A F F L T L K Y A L G T K W S P L S L A A F T G E L T K L R S L M	300
genG_NY	R R Q G L L S N R Y Y A M V G D I G K Y I E N S G L T A F F L T L K Y A L G T K W S P L S L A A F T G E L T K L R S L M	300
genG_Iowa	R R Q G L L S N R Y Y A M V G D I G K Y I E N S G L T A F F L T L K Y A L G T K W S P L S L A A F T G E L T K L R S L M	300
genH	R R Q G L L S N R Y Y A M V G D I G K Y I E N S G L T A F F L T L K Y A L G T K W S P L S L A A F T G E L T K L R S L M	300
JL2	M L Y R D L G E Q A R Y L A L L E A P Q I M D F A P G G Y P L I F S Y A M G V G T V L D V Q M R N Y T Y A R P F L N G Y	360
JL5	M L Y R G L G E Q A R Y L A L L E A P Q I M D F A P G G Y P L I F S Y A M G V G T V L D V Q M R N Y T Y A R P F L N G Y	360
genG_NY	M L Y R D L G E Q A R Y L A L L E A P Q I M D F A P G G Y P L I F S Y A M G V G T V L D V Q M R N Y T Y A R P F L N G Y	360
genG_Iowa	M L Y R D L G E Q A R Y L A L L E A P Q I M D F A P G G Y P L I F S Y A M G V G T V L D V Q M R N Y T Y A R P F L N G Y	360
genH	M L Y R D L G E Q A R Y L A L L E A P Q I M D F A P G G Y P L I F S Y A M G V G T V L D V Q M R N Y T Y A R P F L N G Y	360
JL2	Y F Q I G V E T A R R Q Q G T V D N R V A D D L G L T P E Q R T E V T Q L V D R L A R G R G A G I P G G P V N P F V P P	420
JL5	Y F Q I G V E T A R R Q Q G T V D N R V A D D L G L T P E Q R T E V T Q L V D R L A R G R G A G I P G G P V N P F V P P	420
genG_NY	Y F Q I G V E T A R R Q Q G T V D N R V A D D L G L T P E Q R T E V T Q L V D R L A R G R G A G I P G G P V N P F V P P	420
genG_Iowa	Y F Q I G V E T A R R Q Q G T V D N R V A D D L G L T P E Q R T E V T Q L V D R L A R G R G A G I P G G P V N P F V P P	420
genH	Y F Q I G V E T A R R Q Q G T V D N R V A D D L G L T P E Q R T E V T Q L V D R L A R G R G A G I P G G P V N P F V P P	420
JL2	V Q Q Q Q P A A V Y E D I P A L E E S D D D G D E D R G A G F Q N G V Q V P A V R Q G G Q T D F R A Q P L Q D P I Q A Q	480
JL5	V Q Q Q Q P A A V Y E D I P A L E E S D D D G D E D G G A G F Q N G V Q L P A V R Q G G Q T D F R A Q P L Q D P I Q A Q	480
genG_NY	V Q Q Q Q P A A V Y E D I P A L E E S D D D G D E D G G A G F Q N G A Q A P A V R Q G G Q N D F R A Q P L Q D P I Q A Q	480
genG_Iowa	V Q Q Q Q P A A V Y E D I P A L E E S D D D G D E D G G A G F Q N G A Q A P A V R Q G G Q N D F R A Q P L Q D P I Q A Q	480
genH	V Q Q Q Q P A A I Y E D I P A L E E S D D D G D E D G G A G F Q N G A Q A P A V R Q G G Q N D F R A Q P L Q D P I Q A Q	480
JL2	L F M P L Y P Q V S N I P N N Q N H Q I N R I G G L E N Q D L L R Y N E N G D S Q Q D A R G E H G N T F P N N P N Q N A	540
JL5	L F M P L Y P Q V S N M P N N Q N H Q I N R I G G L E H Q D L L R Y N E N G D S Q Q D A R G E H V N T F P N N P N Q N A	540
genG_NY	L F M P L Y P Q V S N I P N H Q N H Q I N R I G G M E H Q D L L R Y N E N G D S Q Q D A R G E H G N T F P N N P N Q N A	540
genG_Iowa	L F M P L Y P Q V S N I P N H Q N H Q I N R I G G M E H Q D L L R Y N E N G D S Q Q D A R G E H G N T F P N N P N Q N A	540
genH	L F M P L Y P Q V S N I P T H Q N H Q I N R V G G M E H Q D L L R Y N E N G D S Q Q D A R G E H G N T F P N N P N Q N A	540
JL2	Q L Q V G D W D E	549
JL5	Q L Q V G D W D E	549
genG_NY	Q S Q V G D W D E	549
genG_Iowa	Q S Q V G D W D E	549
genH	Q S Q V G D W D E	549
